# Ultra-fast self-assembly and stabilization of reactive nanoparticles in reduced graphene oxide films

**DOI:** 10.1038/ncomms12332

**Published:** 2016-08-12

**Authors:** Yanan Chen, Garth C. Egan, Jiayu Wan, Shuze Zhu, Rohit Jiji Jacob, Wenbo Zhou, Jiaqi Dai, Yanbin Wang, Valencia A. Danner, Yonggang Yao, Kun Fu, Yibo Wang, Wenzhong Bao, Teng Li, Michael R. Zachariah, Liangbing Hu

**Affiliations:** 1 Department of Materials Science and Engineering, University of Maryland College Park, 1208 Engineering Lab Building, College Park, Maryland 20742, USA; 2 Department of Chemical and Biomolecular Engineering, University of Maryland College Park, College Park, Maryland 20742, USA; 3 Department of Chemistry and Biochemistry, University of Maryland College Park, College Park, Maryland 20742, USA; 4 Department of Mechanical Engineering, University of Maryland College Park, College Park, Maryland 20742, USA

## Abstract

Nanoparticles hosted in conductive matrices are ubiquitous in electrochemical energy storage, catalysis and energetic devices. However, agglomeration and surface oxidation remain as two major challenges towards their ultimate utility, especially for highly reactive materials. Here we report uniformly distributed nanoparticles with diameters around 10 nm can be self-assembled within a reduced graphene oxide matrix in 10 ms. Microsized particles in reduced graphene oxide are Joule heated to high temperature (∼1,700 K) and rapidly quenched to preserve the resultant nano-architecture. A possible formation mechanism is that microsized particles melt under high temperature, are separated by defects in reduced graphene oxide and self-assemble into nanoparticles on cooling. The ultra-fast manufacturing approach can be applied to a wide range of materials, including aluminium, silicon, tin and so on. One unique application of this technique is the stabilization of aluminium nanoparticles in reduced graphene oxide film, which we demonstrate to have excellent performance as a switchable energetic material.

## Abstract (web summary)

Nanoparticles of non-noble metals are useful for many applications but can be prone to sintering or surface oxidation. Here, the authors use a rapid heating-cooling approach to synthesize uniformly distributed nanoparticles in a reduced graphene oxide matrix, and test them as switchable energetic materials.

## Graphical abstract

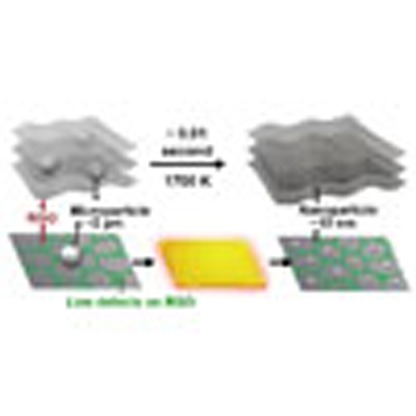

From the Lycurgus cup as early as the fourth century to modern cancer sensing and energy storage, nanoparticles are widely used in physical^1^,^2^,^3^, chemical^4^,^5^,^6^, environmental^7^,^8^, biological^9^,^10^ and medical applications^11^,^12^, owing to their interesting size-dependent properties. Numerous scalable and low-cost methods for making high-quality nanoparticles have been reported and many types of nanoparticles are commercially available^13^,^14^. These synthesis methods can be generally classified into two categories. The first category features ‘top-down' approaches, usually solid-phase methods, including ball milling^15^,^16^, pulsed laser ablative deposition^17^,^18^,^19^, exploding wire^13^,^20^,^21^ and so on. The second category features ‘bottom-up' approaches, such as chemical and physical vapour deposition^22^,^23^ and liquid-phase chemical synthesis^24^,^25^,^26^,^27^,^28^,^29^,^30^,^31^,^32^,^33^. Although many of these approaches have been quite successful, there are significant challenges that still exist. This is especially true for non-noble metals that can be highly reactive and tend to suffer from surface oxidation and agglomeration, which can significantly constrain nanoparticle functionality^34^,^35^,^36^. A fast, low-cost and scalable approach to manufacturing uniformly distributed nanoparticles, especially for energetic nanoparticles, without these impairments is urgently needed. At the same time, nanoparticles hosted in a conductive matrice are ubiquitous in electrochemical energy storage, catalysis and energetic devices, where a conductive matrix can provide fast electron transport for the operations of the devices. Usually, nanoparticles are synthesized first and then assembled into a conductive carbon matrix^37^. For reactive nanoparticles, significant oxidation often occurs during the two steps, which presents a major challenge to the effective use of these nanoparticles^38^.

Here we report an ultra-fast (as fast as 2 ms) process to produce uniformly distributed nanoparticles in a conductive reduced graphene oxide (RGO) matrix by directly Joule heating a metal/semiconductor-RGO film (Fig. 1a) to a high temperature (∼1,700 K or higher). Microsized metal or semiconductor particles used as precursors are melted under high-temperature Joule heating, then self-assemble into nanoparticles on cooling. Agglomeration and coalescence of the nanoparticles, which is driven by surface energy minimization, is suppressed by the defects of RGO in-plane and RGO layers out-of-plane (Fig. 1b). These defects serve as barriers for atom migration, to keep the uniformly distributed nanoparticles separated. The particles within the matrix during heating are protected from oxidation by the RGO sheets, owing to the impermeability of RGO to O_2_ and H_2_O^39^. Thus, RGO sheets serve as a perfect host material for such unique high-temperature process, owing to their defect sites and high melting temperature (stable up to 3,300 K)^40^,^41^,^42^,^43^,^44^,^45^. Furthermore, this high-temperature process for *in situ* synthesis of nanoparticles without agglomeration and surface oxidation is applicable to any materials with a lower melting point than 3,300 K. In this study, we demonstrate fast nanoparticle formation for aluminium (Al), silicon (Si), tin (Sn), gold (Au) and palladium (Pd). These materials in their nanoparticle form have been widely used in energetics, energy storage, optical, sensing and catalytic applications. We choose nAl-RGO to demonstrate its superior properties as an energetic material. Furthermore, we combined three-dimensional (3D) printing with this technique for both precise shape control and scalable manufacturing of nanoparticles-RGO architectures (Fig. 1c).

## Results

### Synthesis of nanoparticles in RGO network

**Figure 2:**
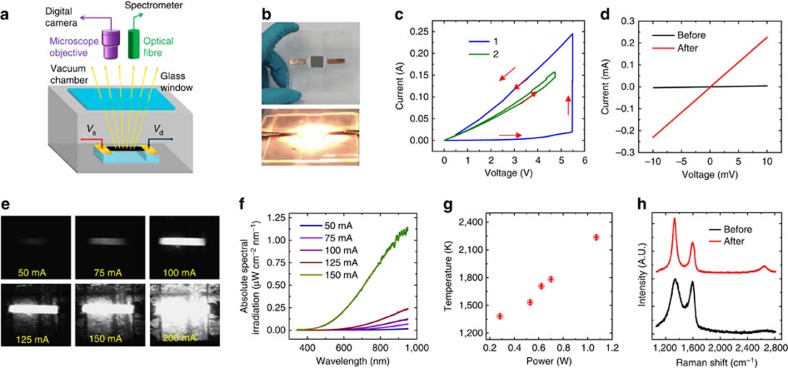
Characterizations of fast nanoparticle formation process. (**a**) Home-made optical measurement setup for metal-RGO high-temperature treatment under an electrical bias in vacuum chamber. (**b**) Photo image of a 2.5 × 2.5 cm Al-RGO film suspended by a glass holder before and during Joule heating treatment. (**c**) *I–V* curve of an Al-RGO film (width: 506 μm, length: 636 μm) during a high-temperature treatment process. (**d**) *I–V* curve of Al-RGO film before and after treatment. (**e**) A sequence of photo images to show the Al-RGO film (width: 380 μm, length: 1917 μm) under Joule heating with an increasing applied electrical power. (**f**) Light emission spectra of Al-RGO film at different current densities. (**g**) Temperature of Al-RGO film at different power. (**h**) Raman spectroscopy of Al-RGO film before and after high-temperature treatment.

The nanoparticle-RGO films were fabricated with a homemade setup, where *in situ* monitoring of the temperature and electrical conductivity during Joule heating can be achieved (Fig. 2a). To obtain the nanoparticle-RGO structure, we started with microsized particles embedded within an RGO thin film prepared by vacuum filtration (Supplementary Fig. 1 and Supplementary Methods). The films were fixed on a glass holder with current applied to achieve high temperature in a vacuum chamber (Supplementary Fig. 2). A typical freestanding Al-RGO film before and during Joule heating is shown in Fig. 2b. The Joule heating process to high temperatures further reduced the RGO^46^,^47^,^48^, leading to a large conductivity increase in the Al-RGO film. Two detailed voltage sweeps of the first and second cycle of the film are shown in Fig. 2c. In the first heating process, the conductivity of Al-RGO film increases more than 100 times (from 1.3 to 139.1 S cm^−1^) on Joule heating. After cooling down to room temperature (Fig. 2d), the conductance of the film was 75 S cm^−1^. In the high-temperature treatment process, a sequence of photo images with increasing applied electrical power is shown in Fig. 2e. To monitor the temperature of the Al-RGO sample, its emitted radiation was recorded *in situ* by an optical fibre and spectrometer. The emission spectrum (Fig. 2f) of the Al-RGO device was then fitted to the Blackbody radiation equation, to extract the temperature of the film (Supplementary Fig. 3 and Supplementary Methods). In the Al-RGO experiment, we obtained different temperatures of 1,380, 1,530, 1,710, 1,780 and 2,200 K at 50, 75, 100, 125 and 150 mA, respectively (Fig. 2g). To demonstrate structural changes, Raman spectra of the Al-RGO films before and after 2,200 K treatments were obtained (Fig. 2h). We observed a largely increased intensity ratio of D (1,350 cm^−1^) to G peak (1,600 cm^−1^) (*I*_D_/*I*_G_), from 1.03 to 1.83, after Joule heating. The Al-RGO film maintains a high *I*_D_/*I*_G_ ratio in the high-temperature treatment process (Supplementary Fig. 4 and Supplementary Note 1), which is strikingly different with that of pure RGO films (Supplementary Fig. 5). The difference in Raman spectrum is possibly correlated with the formation of Al nanoparticles. As we believe the nanoparticles are stabilized by the defect sites, the defect density is thus stabilized by the nanoparticles such that the defect density is constant in Al-RGO^49^, whereas the defects in pure RGO film are self-healed at high temperature^50^.

### Characterization of nanoparticles in RGO network

**Figure 3:**
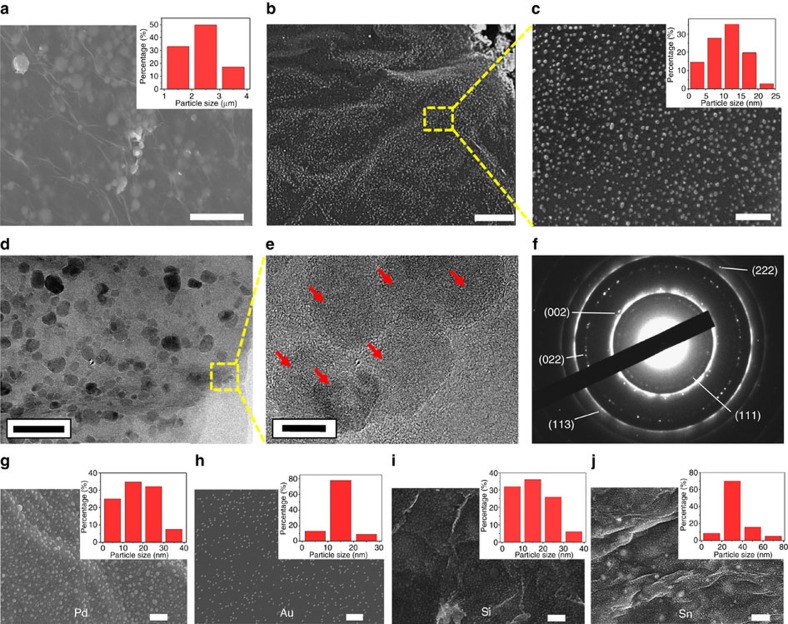
Characterization of nanoparticles formed in RGO network. (**a**) SEM image of RGO films with embedded Al microsized particles. (**b**,**c**) SEM images of Al nanoparticles after 2,200 K Joule heating for 1 min. (**d**–**f**) TEM images of nAl-RGO with different magnifications and the corresponding selected area diffraction pattern patterns. (**g**–**j**) SEM images of nanoparticles formed in RGO network with Pd, Au, Si and Sn. Particle size distribution are also shown in the insets. Scale bar, (**a**) 10 μm, (**b**) 1 μm, (**c**) 200 nm, (**d**) 100 nm, (**e**) 10 nm, (**g**,**h**) 200 nm, (**i**) 300 nm and (**j**) 1 μm.

To understand the formation of nanoparticles during high-temperature treatment, scanning electron microscopy (SEM) and transmission electron microscopy (TEM) are applied to characterize the Al-RGO before and after Joule heating treatment at 2,200 K for 1 min. The Al particles in RGO film before treatment have an average diameter of 2 μm (Fig. 3a). After treatment, high-density Al nanoparticles were dispersed uniformly in RGO layers (Fig. 3b,c), with sizes ranging from 1.0 to 25.0 nm (Fig. 3c, inset). The interior structure (Supplementary Fig. 6) and the cross-sectional morphology of Al-RGO film (Supplementary Fig. 7) confirm high-density distribution of Al nanoparticles within the entire RGO network. The rings of distinct spots in the selected area diffraction pattern indicate polycrystalline structure (Fig. 3d–f), which results from multiple nanoparticles in the electron diffraction area. The spacing of these rings is consistent with face-centered cubic (FCC) Al. The brighter, continuous rings agree with graphite. It is noteworthy that this particular sample was exposed to air before TEM analysis; however, from the diffraction pattern, we did not observe the formation of Al_2_O_3_. This result indicates that the RGO can serve as an oxidation barrier for zero-valent metals. We also demonstrated that the proposed heating method for metal nanoparticles synthesis can be applied to a broad range of materials, such as Pd, Au, Si and Sn (Fig. 3g–j and Supplementary Methods).

### Ultra-fast synthesis of Al nanoparticles

**Figure 4:**
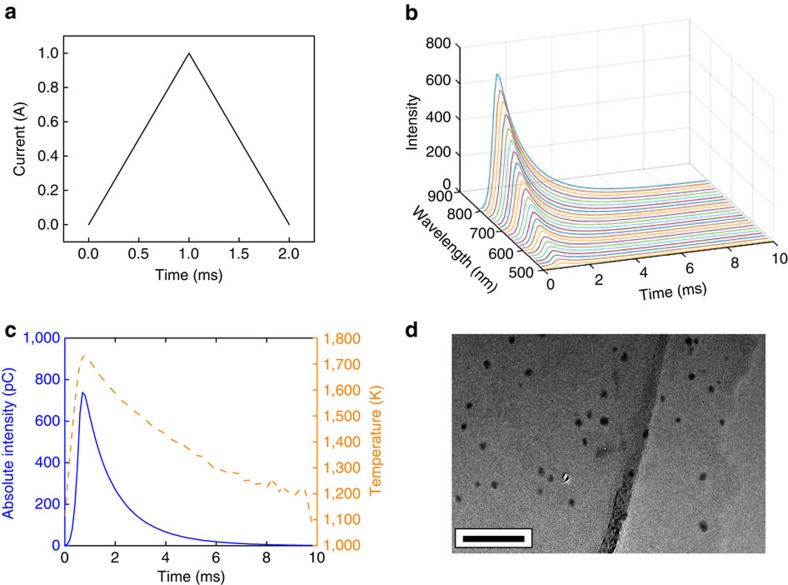
Characterization of ultra-fast Al nanoparticles formation process. (**a**) Current value programmed for the high-speed spectroscopic temperature measurement for Al-RGO sample. (**b**) Emission spectra of Al-RGO film in the ultra-fast heating process. (**c**) Temperature and intensity profile on the 800 nm channel in the ultra-fast heating process. (**d**) TEM image of Al nanoparticles formed in RGO network after ultra-fast heating. Scale bar, 50 nm (**d**).

The longer a material is heated, the more significant heat losses become. Therefore, to make this process as energy efficient as possible, it is important to quantify the minimal time needed to achieve nanoparticle synthesis. To this end, we quantified the high-temperature self-assembly of Al nanoparticles in RGO matrix driven by current applied for only 2 ms (Supplementary Figs 8 and 9). The fast current pulse through the Al-RGO sample (1,730 μm long, 960 μm wide and 4 μm thick) was programed to go from 0 to 1.05 A within 1 ms (Fig. 4a). The materials response was monitored with a specially designed sub-millisecond diagnostics (Supplementary Fig. 10 and Supplementary Note 2). The output spectra from the 24 channels plotted versus time with the wavelength axis shows the central wavelength of each channel (Fig. 4b). The intensity peaks occur within the millisecond time frame. The time-resolved spectrum was used to extract the temperature (Fig. 4c) accompanied by the intensity profile on the 800 nm channel. The temperature rise time and cooling time were estimated to be ∼900 μs and ∼9 ms, respectively, with the cooling occurring after Joule heating via conduction to the holder and radiation to the surroundings (no convection due to the experiments being done under vacuum). As the photo multiplier tube (PMT) operated in the visible range, the lowest temperatures it could detect with reasonable accuracy was around 1,000 K and, as the intensity levels dropped with the sample cooling down, the signal-to-noise ratio was substantially reduced resulting in inaccurate temperature prediction at the later stages. The synthesis process of Al nanoparticles is around 10 ms, which is much faster than many traditional synthesis methods for nanoparticles, such as ball milling, physical vapour deposition and liquid-phase chemical synthesis. The highest temperature achieved for this nAl-RGO sample was 1,730 K in the ultra-fast heat process. After cooling down, the obtained Al nanoparticles have an average size of 10 nm. A typical morphology is shown in Fig. 4d.

### Formation mechanism of nanoparticles in RGO

We propose the following mechanism for the ultra-fast, *in situ* nanoparticles formation at high temperatures. Al microsized particles melt under Joule heating and presumably disperse throughout the films. As cooling takes place, the nucleation occurs around the defects on the RGO network and accretes Al atoms to form ultra-fine nanoparticles. We have noticed that the temperature involved in our heating may be beyond the bulk boiling point of aluminium. To this regard, we cannot exclude the possibility of aluminium vapourization in the high-temperature treatment process. However, owing to the encapsulation effect of RGO layers, even if there are vapourized aluminium atoms, they will remain in between RGO layers. Next, during the cooling process, these vapourized aluminium atoms can re-group and condense into new nanoparticles. Therefore, such mechanism is in-effect similar to melting, but with an intermediate vapourizing and condensation stage, before reshaping into new nanoparticles. This is an interesting topic to investigate in future studies. A striking feature of the resulting nanoparticles is their superior structural stability. One ubiquitous challenge to nanoparticles is their tendency to merge and agglomerate at high temperature, driven by surface energy minimization. However, the nanoparticles formed at 2,200 K in our experiments remain stable without appreciable merging or agglomeration. We attribute the superior stability of the resulting nanoparticles to a barrier effect of the defects in RGO, as to be shown by atomistic modelling analysis. Defects (for example, vacancies, grain boundaries and voids) inevitably form during the synthesis of RGO. We investigate the effect of defects in RGO on the dispersion of nanoparticles and find that the defects can serve as an effective barrier so that the migration of nanoparticles is constrained to be within the domain demarcated by neighbouring defects. As a result, nanoparticles remain isolated from each other and thus no agglomeration and merging occur.

**Figure 5:**
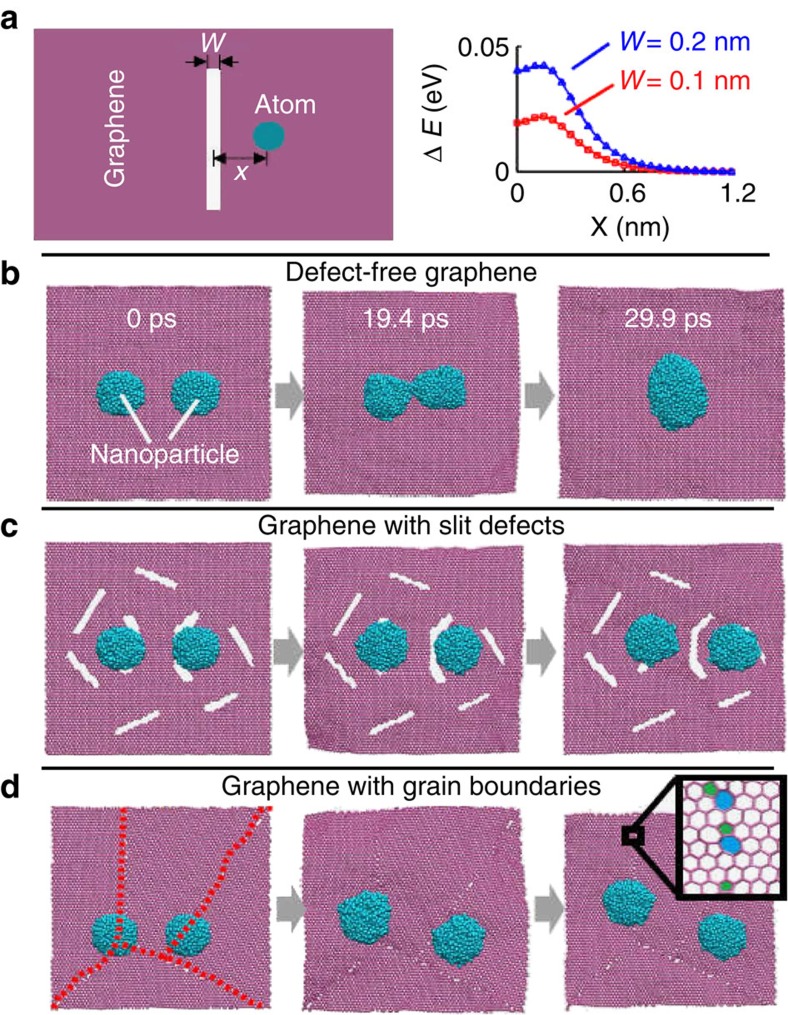
Barrier effect of defects on structural stability of nanoparticles. (**a**) Schematic of an atom located with a distance of *x* to the center of a slit defect of width *w* in graphene and the evolution of potential energy as the function of *x*, clearly showing an energy barrier as the atom approaches the defect. (**b**) On defect-free graphene, two neighbouring 3-nm Al nanoparticles are initially apart from each other by 5 nm, then migrate by Brownian random walk at 2,200 K and eventually aggregate and coalesce into a single particle, driven by surface energy minimization. (**c**) On graphene with slit defects, two 3 nm Al nanoparticles with the same initial dispersion distance are shown to be effectively confined within the domain demarcated by the slit defects, owing to the barrier effect. The two nanoparticles remain dispersed without coalescence at 2,200 K. (**d**) On graphene with grain boundaries (as outlined by dotted lines in left panel, mainly made of pentagon-heptagon paired defects), the two nanoparticles also remain dispersed without coalescence at 2,200 K.

We first demonstrate the barrier effect of the defects (for example, an open slit) on a single foreign atom (for example, an Al atom) through a theoretical model (Fig. 5a, Supplementary Fig. 11 and Supplementary Note 3). As shown in the right panel of Fig. 5a, the potential energy of the system reaches its minimum as the Al atom is far away from the defect. As the Al atom approaches the slit defect, the total potential energy of the system increases, peaks near the edge of the slit defect and then decreases slightly as it approaches the centre of slit defect width. In other words, there exists an energy barrier for the Al atom to migrate across the slit defect and a local minimum that could pin the atom at the defect. The energy barrier increases as the width of the slit defect increases. As such an energy barrier essentially results from the change of atom density at the defect site^51^ (that is, missing atoms from a perfect material lattice structure), the barrier effect of defects is generally applicable to various defect types and foreign atom species^51^. We next perform molecular dynamics (MD) simulations, to demonstrate that such a defect-induced energy barrier can effectively constrain the migration of nanoparticles and thus prevent them from merging at a high temperature. We begin our MD studies with a control simulation (Fig. 5b), on defect-free graphene, two 3 nm Al nanoparticles are initially located 5 nm apart from each other. Owing to thermal fluctuations at 2,200 K, the Al nanoparticles will experience Brownian random walk and, when close enough to experience the weak van der Waals forces, eventually aggregate and coalesce to minimize the surface energy (Supplementary Movie 1). In contrast, if two 3 nm Al nanoparticles are dispersed with the same distance on graphene with slit defects (Fig. 5c), the random walk of each nanoparticles is effectively confined within a domain demarcated by neighbouring defects, due to the barrier effect. As a result, the two nanoparticles remain dispersed without aggregation and coalescence (Supplementary Movie 2). Figure 5d further demonstrates that graphene boundaries can also serve as an effective barrier to confine the thermo-activated random walk of the nanoparticles and thus prevent the coalescence of neighbouring nanoparticles (Supplementary Movie 3). Furthermore, as the chemical reactivity of the carbon atoms at the defect sites is relatively higher than those away from defects, the chemical bonding between these carbon atoms and the nanoparticles could also possibly occur^52^, which could further anchor the nanoparticles and prevent them from merging at high temperature.

### Al nanoparticles-RGO as switchable energetic material

Among their many potential applications, these well-dispersed nanoparticles without surface oxidation in a conductive matrix are particularly well suited for use as energetic materials, which is a class of materials defined by the ability to very rapidly convert stored chemical potential energy to thermal energy, to produce work (for example, propellants and explosives). There has been a recent drive to employ nanoparticles in such applications, because they have been demonstrated to burn faster and have lower ignition temperatures than their microsized counterparts^53^,^54^,^55^,^56^. However, reactive nanoparticles are also likely to agglomerate and to suffer rapid loss of surface area due to coalescence and sintering on heating, which diminishes the advantages of using nanoparticles (that is, enhanced specific surface area and shorter diffusion distances)^57^,^58^,^59^. Native oxide layers are another issue as even the typical 2–5 nm layer that forms on contact with air can represent a significant portion (∼30%) of a nanoparticle's mass^53^. In addition, one major practical concern for nanoenergetic materials is safety, particularly in regards to avoiding unplanned ignition event. Thus, an ideal energetic system would stabilize the nanostructure against agglomeration and oxidation so the full potential of the nanoscale could be realized, while being insensitive to accidental ignition. Here we demonstrate that the nAl-RGO produced in this study achieves much of the ideality outlined above.

**Figure 6:**
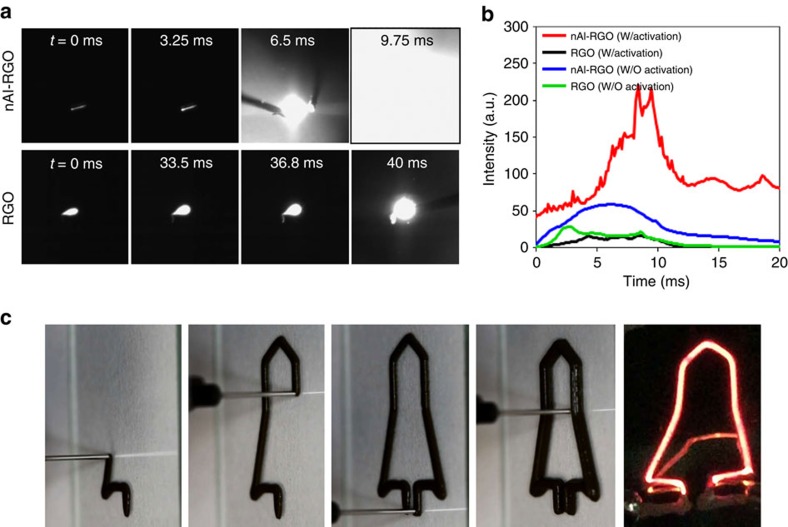
Application of Al nanoparticles in RGO network as an energetic material. (**a**) Frames from high-speed videos with 142 μs exposure time of activated nAl-RGO and RGO that werekept hot and flooded with 3 atm O_2_. Time 0 for both materials was arbitrarily defined and the last frame shown is the one with peak integrated intensity. (**b**) The normalized integrated intensity taken from the video frames (20 μs exposure) of nAl-RGO and RGO samples that were activated (as in **a**) and that were subject to direct heating in 3 atm of O_2_ without activation. (**c**) Thee-dimensional printing process of Al-RGO and *in situ* formation to nAl-RGO by fast Joule heating.

The energetic nature of this material is illustrated in Fig. 6. The first line of images in Fig. 6a shows frames from high-speed video of activated 1:1 nAl-RGO that was flooded with O_2_ to produce a violent reaction. The sample (1,700 μm long, 410 μm wide and 4 μm thick) was connected to electrical leads with silver paste and heating was achieved by passing current (∼70 mA) through the material. Before reaction, this sample was held at a temperature (∼1,500 K based on previous pyrometry results) for 2 min in a chamber pumped to 0.001 atm, to activate it by allowing for reduction of extraneous Al_2_O_3_ on the outer surface of RGO (see discussion below). Following activation, the chamber was then opened to 3 atm of pure O_2_, while the material was still hot. The second row of images in Fig. 6a shows the reaction of RGO alone under the same conditions, which was far less violent and thus illustrates the immense enhancement achieved by adding Al nanoparticles to the system.

Reactivity is quantified in Fig. 6b by plotting the temporal emission intensity taken from the individual frames of high-speed video (frames from the specific videos used to make this plot are shown in Supplementary Fig. 12). The plot shows that RGO alone, both with activation (preheated in vacuum; black line) and without activation (heated directly in 3 atm of O_2_; green line) exhibited similar weak emission. The nAl-RGO film without activation (blue line) shows modest enhanced emission over RGO, indicating the presence of some active Al nanoparticles. By contrast, the activated film material (that is, material that was thermally processed before admission of the oxidizer; red line) shows a greatly enhanced emission event, consistent with the differences seen in Fig. 6a. It is noteworthy that although different heating and oxygen introduction conditions were used for the ‘with activation' and the ‘without activation' samples, the similar performance of the control RGO enables this comparison between the nAl-RGO experiments under different conditions.

Our hypothesis for this observed behaviour is that after the initial synthesis of the nanoparticles, when the film is exposed to air, the Al nanoparticles on the outer surface of the RGO are oxidized, which diminishes the reactivity when the sample is heated directly without activation. During activation, the oxide is reduced by the high temperature and the presence of carbon during activation, which leaves a highly reactive nanofuel that can fully participate in the reaction. Therefore, the RGO matrix provides both an avenue for overcoming nanoparticle oxidation and physical stabilization against aggregation and coalescence. More information on this carbothermic reduction process can be found in Supplementary Fig. 13 and Supplementary Note 4.

In regards to safety, the difference between the two composite cases is evidence that energetic release of this material can be tuned *in situ* in a manner analogous to an ON–OFF switch. Such a capability would greatly enhance the handling of energetic materials and enable the use of materials that would be nominally too dangerous to employ. This concept is further supported by the failure of the non-activated material to burn when heated with a butane torch as shown in Supplementary Fig. 14 and Supplementary Note 5. Another benefit of material is that it can be processed with 3D printing technique. Applications such as micropyrotechnics and micropropulsion are dependent on patterning energetic material with fine control, which is something printing provides^60^. An example of this is shown in Fig. 6c, which demonstrates printing by a 3D printer using liquid GO-Al ink^61^,^62^,^63^. In this case, the *in situ* nanoparticle generation and activation can be done directly by Joule heating of the printed material as demonstrated by the emission in the far right image in Fig. 6c. Experimental details are described in Supplementary Figs 15,16 and Supplementary Note 6.

## Discussion

We report fine nanoparticles with an average size of 10 nm can be formed *in situ* inside a conductive RGO matrix at high temperature. The method is particularly attractive for reactive or energetic nanoparticles. For example, surface-oxidation-free Al nanoparticles are uniformly distributed without any agglomeration. This *in situ* methodology by Joule heating for fast nanoparticle fabrication avoids two typical challenges in active nanoparticle synthesis and assembly, which are surface oxidation and agglomeration due to the large specific surface area. Defect-guided self-assembly is likely to be the underlying mechanism of the superior structural stability of the resulting nanoparticles, supported by MD simulations. Other features that are unique for this *in situ* formation process of nanoparticles in functional structure include the following: (1) synthesis process can be completed within ∼10 ms; (2) the nanoparticles are small with a size around 10 nm; (3) the layered RGO as a barrier can protect the nanoparticles from oxidation; (4) the method can be applicable to a wide range of materials as demonstrated for Sn, Si, Au and Pd. The nAl-RGO composites were demonstrated to have direct applicability as an energetic material with a unique property of switchable reactivity. The RGO matrix was found to enable both initiation of the composites and reduction of extraneous oxide caused by exposure to air. Highly dispersed and oxidation-free, agglomeration-free nanoparticles embedded in a highly conductive matrix can also be used for batteries, catalysts and many other emerging applications.

## Methods

### Materials synthesis

Detailed fabrication processes of various nanoparticles-RGO films are included in the Supplementary Methods.

### Materials characterization

The morphology of the nanoparticles-RGO network was examined by a Hitachi SU-70 field-emission SEM. The SEM images were recorded in secondary electron mode. The samples for SEM surface characterization were prepared directly by adhering the freestanding film to the carbon tape on the stage. The as-prepared freestanding film was broken by tweezers or peeled off by double-sided cellotape for revealing internal surface morphology. TEM JEOL JEM 2100F performed at an accelerating voltage of 200 kV was also employed for the nanoparticle-RGO network morphology characterization. The TEM images were recorded in both high-resolution mode and bright-field mode. For preparing the samples for TEM characterization, the as-prepared nAl-RGO film was added in 1 ml of ethanol followed by sonication for ∼10 h at room temperature until a suspension was achieved. Next, a suspension droplet was applied onto the copper grid. Ten samples for each type of nanoparticles in RGO were fabricated for particles size distribution. Particle size distribution was acquired by statistically analysing a large number of SEM and TEM images using ImageJ software. Raman spectroscopy was performed with a commercial micro Raman spectrometer (Labram Aramis model manufactured by Horiba Jobin Yvon) using a 633 nm He-Ne laser. The thickness of GO nanosheets was determined by atomic force microscopy. Temperature characterizations are included in Supplementary Methods.

### Modelling

We use the large-scale atomic/molecular massively parallel simulator^64^ to perform MD simulations. The non-bonded interaction between carbon atoms in the graphene and the Al atoms in the catalyst nanoparticle is modelled by the Lennard–Jones 12-6 potential 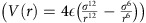 with the parameters 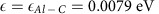 and 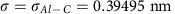, as developed through the customary Lorentz–Berthelot mixing rules, using Al–Al and C–C parameters from the Universal Force Field^65^. We use the embedded atom potential file with the large-scale atomic/molecular massively parallel simulator distribution to describe the bounded interaction between Al atoms in the nanoparticle. The simulation is performed on a canonical ensemble, controlled by a Nosé–Hoover thermostat at 2,200 K. The time step is 0.0001, ps. In all simulations, the Al particle is initially modelled 3 nm in diameter and 5 nm in the distance between centres. The graphene sheet is 15 nm × 15 nm in size.

### Energetic characterization

The reactivity of the samples was characterized using a high-speed camera (Phantom v12.1) and a chamber that could be evacuated to 0.001 atm and maintain a pressure up to 3 atm. The material was connected to electrical leads using silver paste, inserted into the chamber and was Joule heated with ∼70 mA for activation and to drive reaction. Pure O_2_ gas at 3 atm was used as an oxidizer. For the ‘with activation' samples, the material was heated first in vacuum and oxidizer was flowed into the chamber by opening a valve to a pressurized line, while the material was held at high temperature. For the ‘without activation' samples, the chamber was pressurized before heating. It is noteworthy that particular care was taken to keep the sample width, thickness and length constant throughout all experiments to ensure consistent heating conditions.

### Data availability

The data that support the findings of this study are available from the corresponding author upon request.

## Additional information

**How to cite this article:** Chen, Y. *et al*. Ultra-fast self-assembly and stabilization of reactive nanoparticles in reduced graphene oxide films. *Nat. Commun.* 7:12332 doi: 10.1038/ncomms12332 (2016).

## Supplementary Material

Supplementary Information Supplementary Figures 1-16, Supplementary Notes 1-6 and Supplementary Methods.

Supplementary Movie 1 Morphological evolution of Al nanoparticles on defect-free graphene

Supplementary Movie 2 Morphological evolution of Al nanoparticles on graphene with slit defects

Supplementary Movie 3 Morphological migration of Al nanoparticles on graphene with grain boundaries

## Figures and Tables

**Figure 1:**
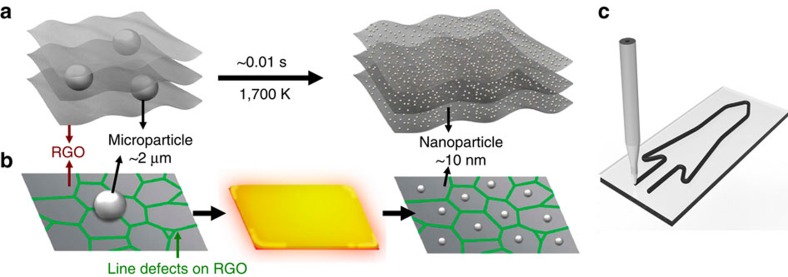
Schematic of *in situ* nanoparticles self-assembly process. (**a**) Microsized particles in a conductive RGO network matrix (left) are self-assembled into nanoparticles (right) driven by direct Joule heating for ∼10 ms. (**b**) Proposed mechanism for nanoparticle formation, where a microsized particle melts on heating and self-assembles into nanoparticles due to confinement by the defects of the RGO sheet. (**c**) The raw materials are 3D printable, where nanoparticles can be formed in a conductive RGO matrix.
