# Supplementary material for: Ultra-fast self-assembly and stabilization of reactive nanoparticles in reduced graphene oxide films
Source: Nat Commun. 2016 Aug 12;7:12332. doi: 10.1038/ncomms12332 (PMC4990634; doi:10.1038/ncomms12332)
Supplement: Supplementary Information — Supplementary Figures 1-16, Supplementary Notes 1-6 and Supplementary Methods. [file ncomms12332-s1.pdf]

Supplementary Figures

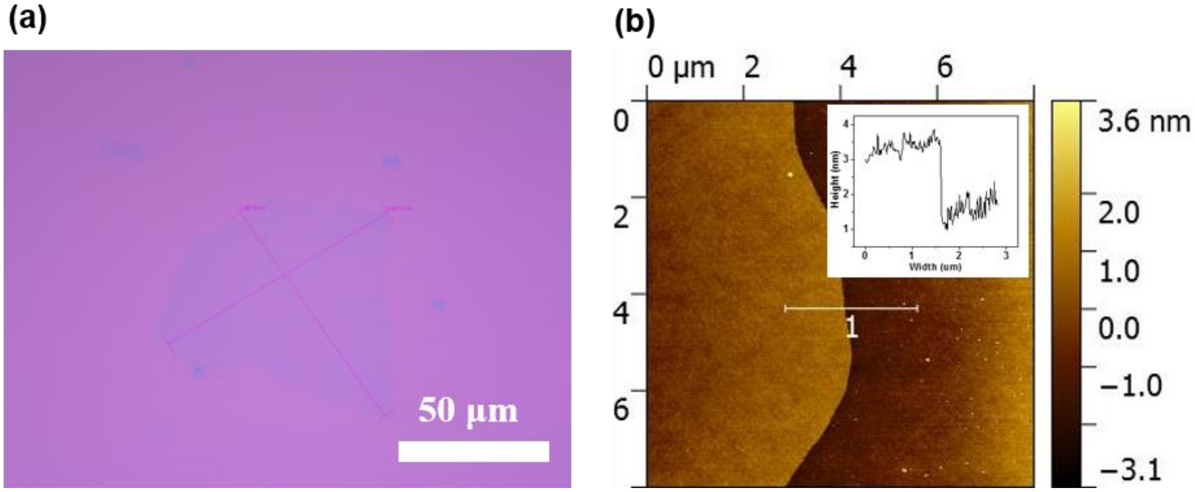

Supplementary Figure 1. Microscope image (a) showing single flake GO and AFM image (b) showing thickness of single flake GO.

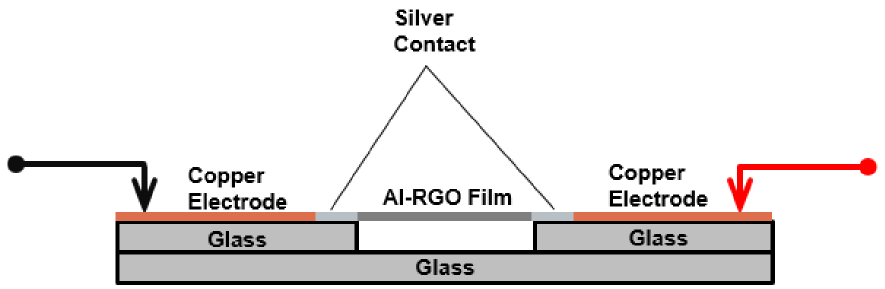

Supplementary Figure 2. Setup for Al-RGO high temperature treatment suspended on a trench formed by glass.

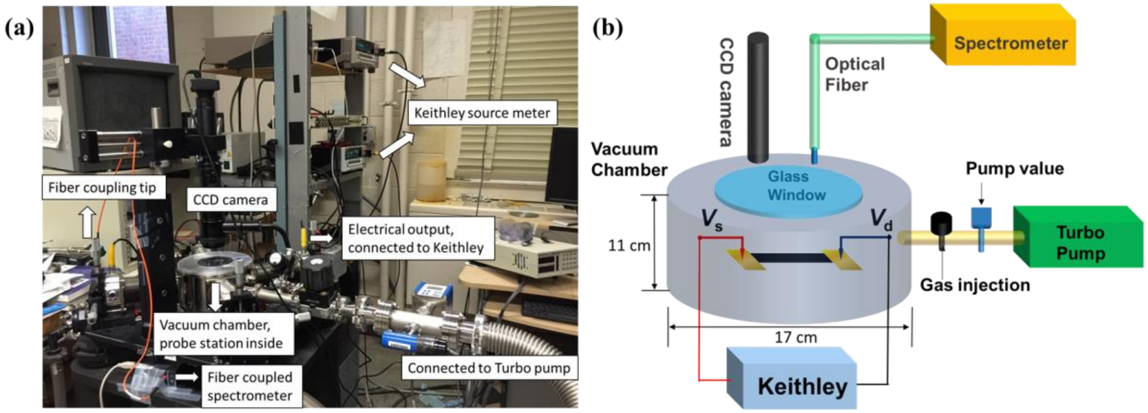

Supplementary Figure 3. Picture and schematic of the home-built setup used for manufacturing Al nanoparticles.

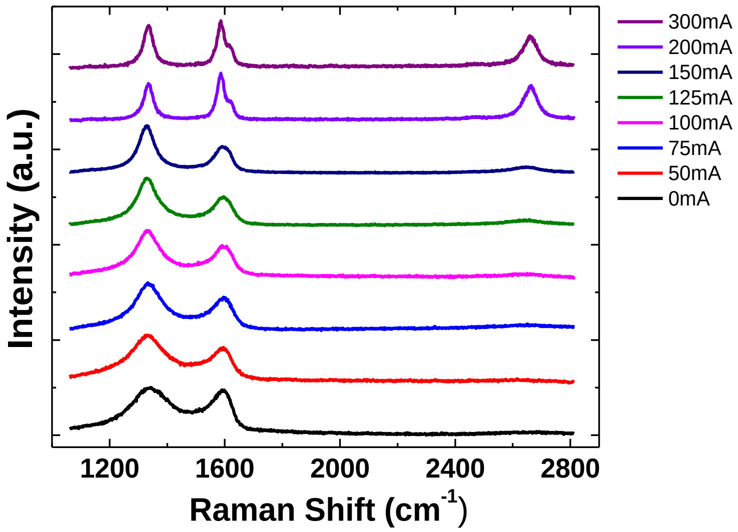

Supplementary Figure 4. Raman spectra for various applied current of the Al-RGO film.

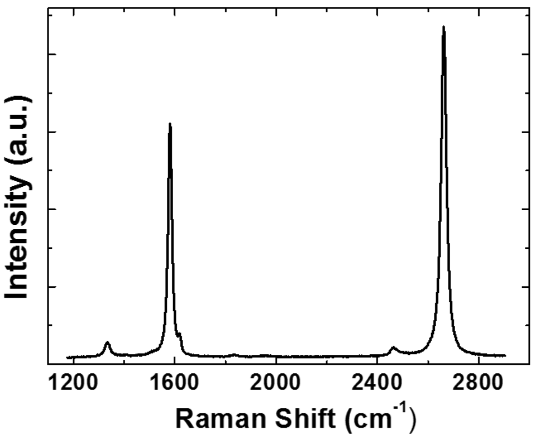

Supplementary Figure 5. Raman spectra of RGO network without Al after 300mA treatment for 1min.

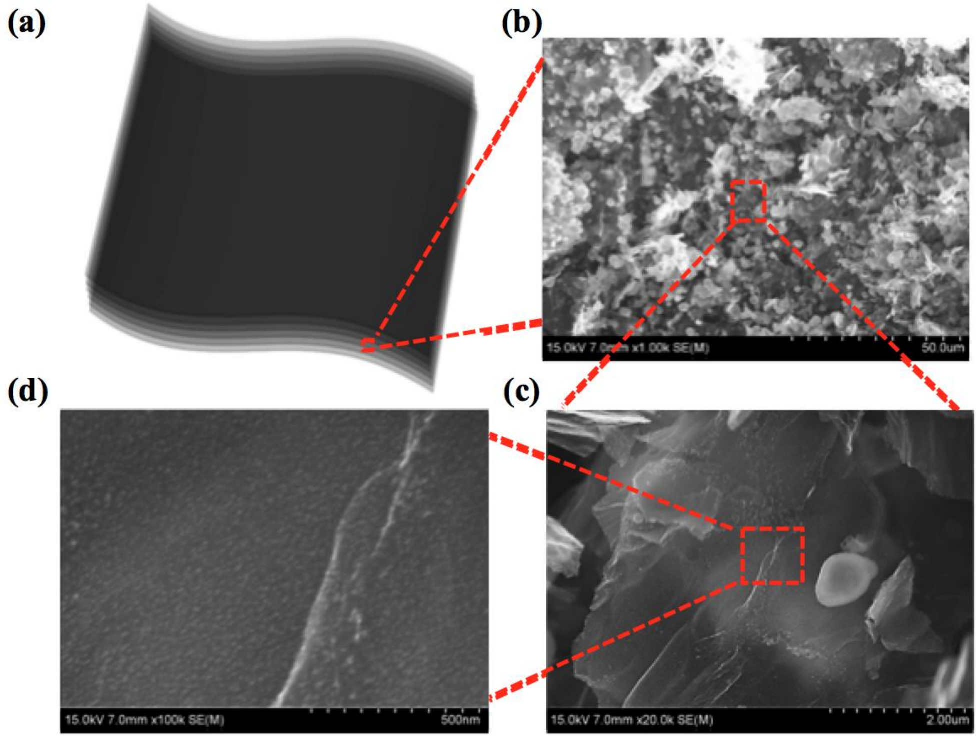

Supplementary Figure 6. SEM images of nAl-RGO film after peeling off the surface, (a) is the schematic, (c) and (d) is zoom-in of (b). The nAl-RGO film was treated at 1400 K for 1 min.

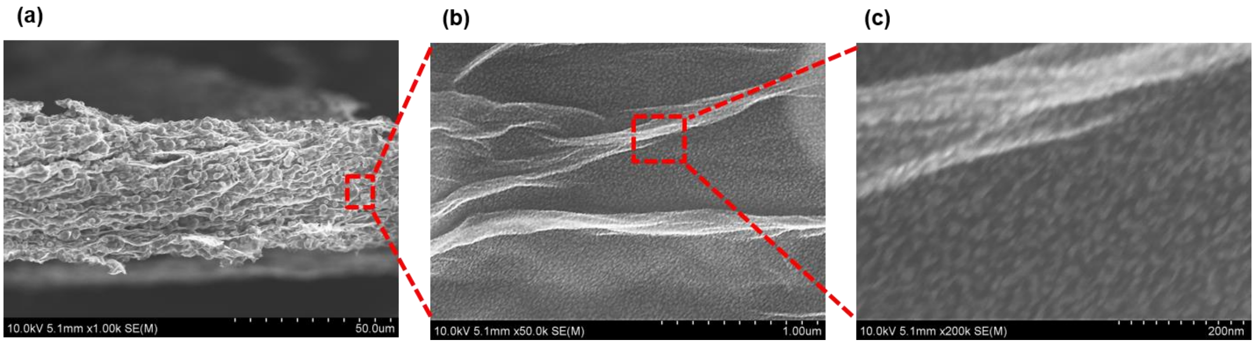

Supplementary Figure 7. Cross-sectional SEM images of nAl-RGO film after high temperature treatment, (b) and (c) is zoom-in of (a). The nAl-RGO film was treated at 1400 K for 1 min.

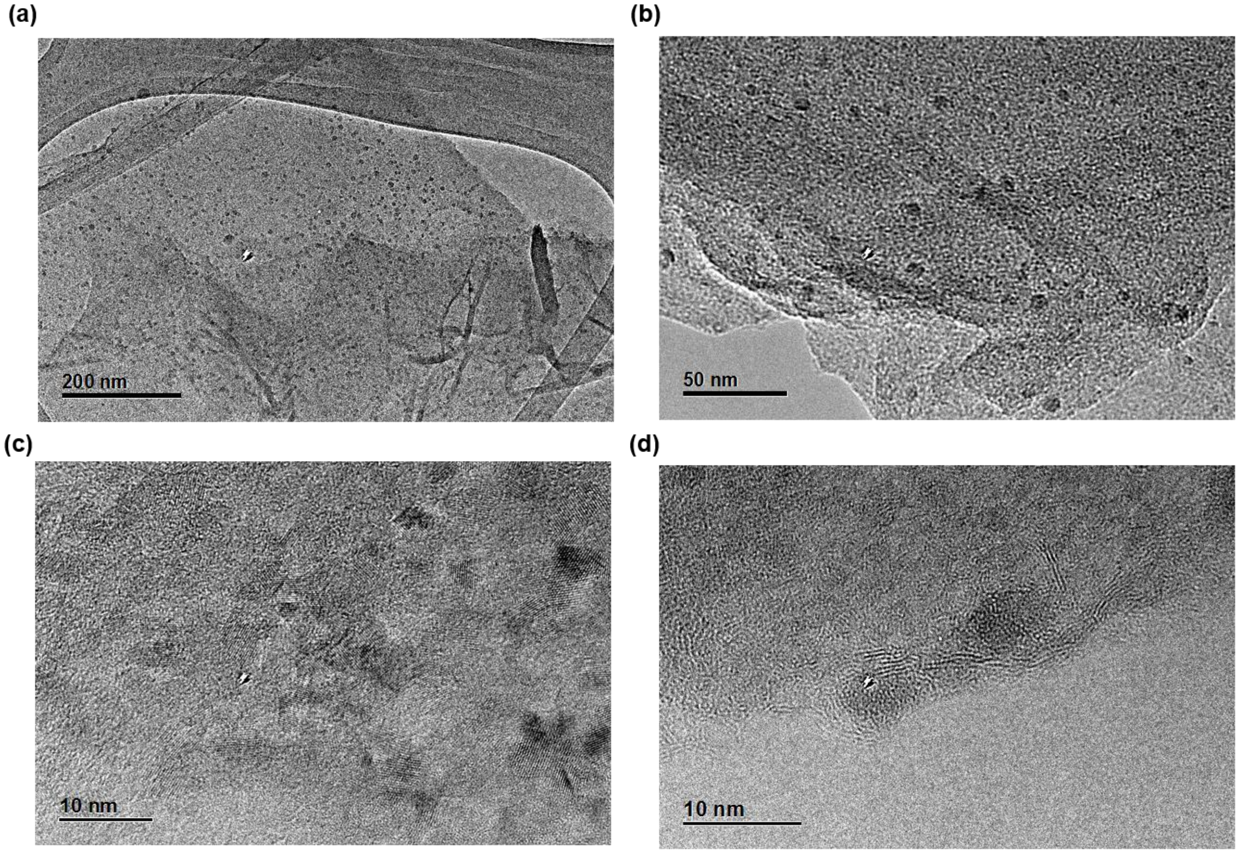

Supplementary Figure 8. TEM images of Al nanoparticles produced in one second with 1.05 A current.

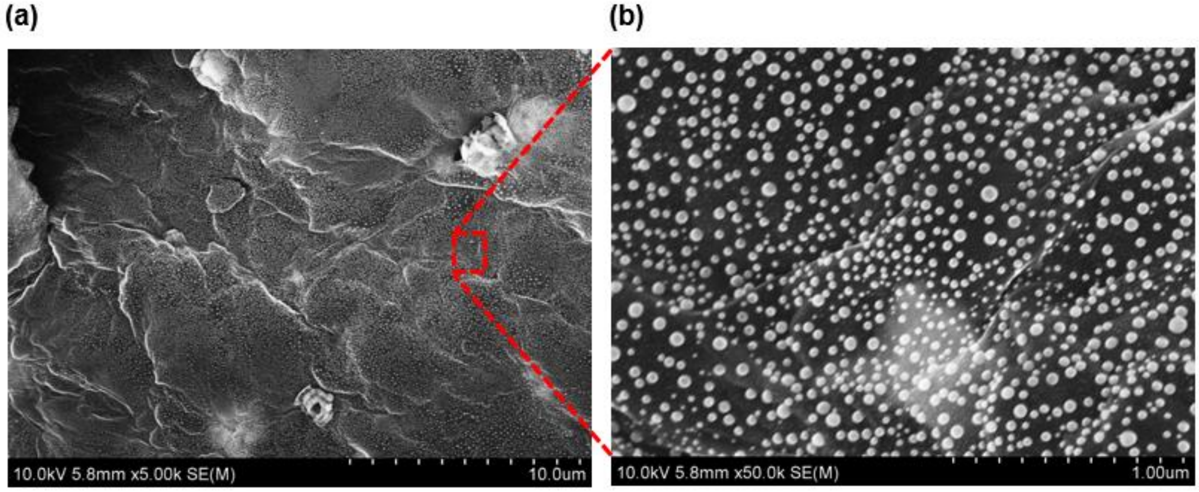

Supplementary Figure 9. SEM images of Al-RGO composite after heating by 1 ms pulse with a current of 1.05 A, (b) is zoom-in of (a). The highest temperature achieved for this sample is around 1350K.

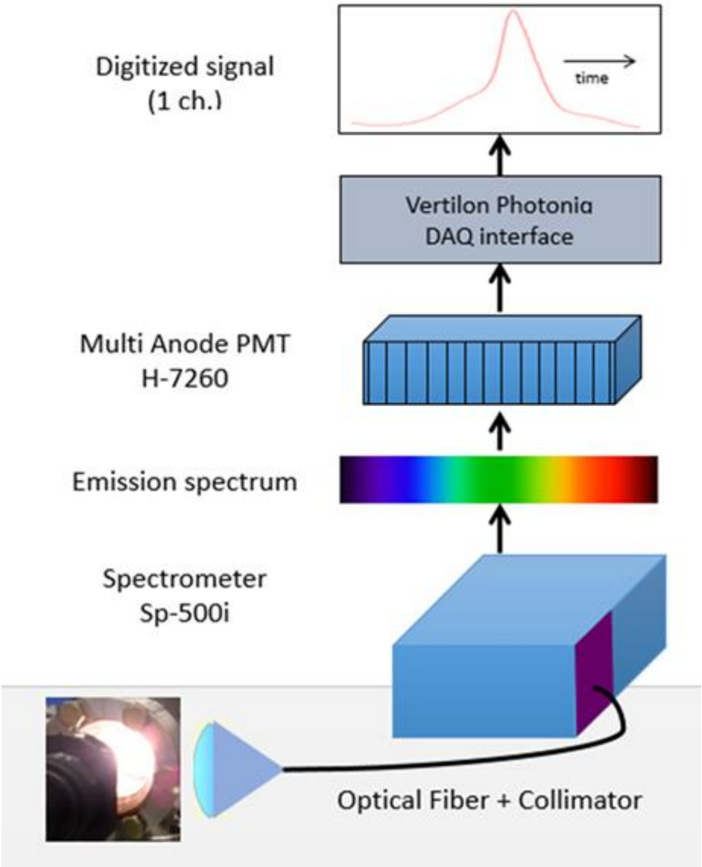

Supplementary Figure 10. Schematic of the setup used for high speed spectroscopic temperature measurement for Al-RGO sample.

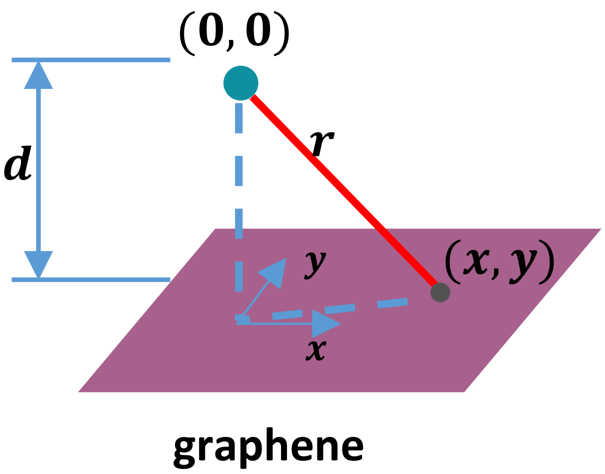

Supplementary Figure 11. A schematic of a foreign atom on a graphene layer with a separation distance of *d*.

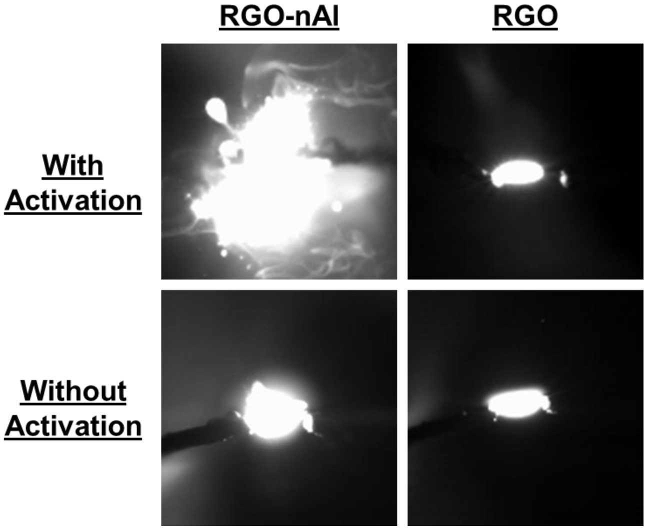

Supplementary Figure 12. Frames with peak integrated intensity from the high speed video used to make Figure 6b in the main text. These videos were taken with 20 μs exposure. Activated samples were heated in vacuum for 1.5 minutes and were ignited by keeping the samples hot and flowing O_2 (3 atm) into the chamber. Samples without activation were heated with a current pulse in a 3 atm O_2 environment.

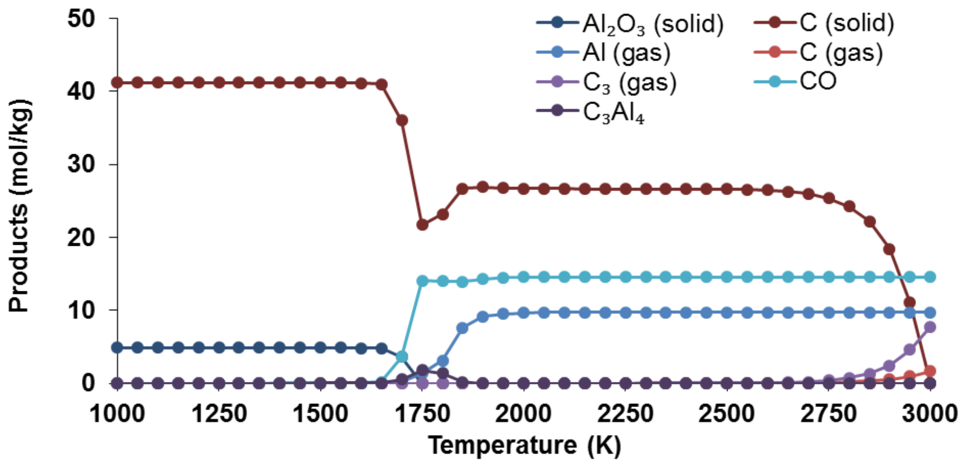

Supplementary Figure 13. Equilibrium calculations made using CHEETAH 6.0 of the products of carbon and alumina. Pressure was held at 0.001 atm.

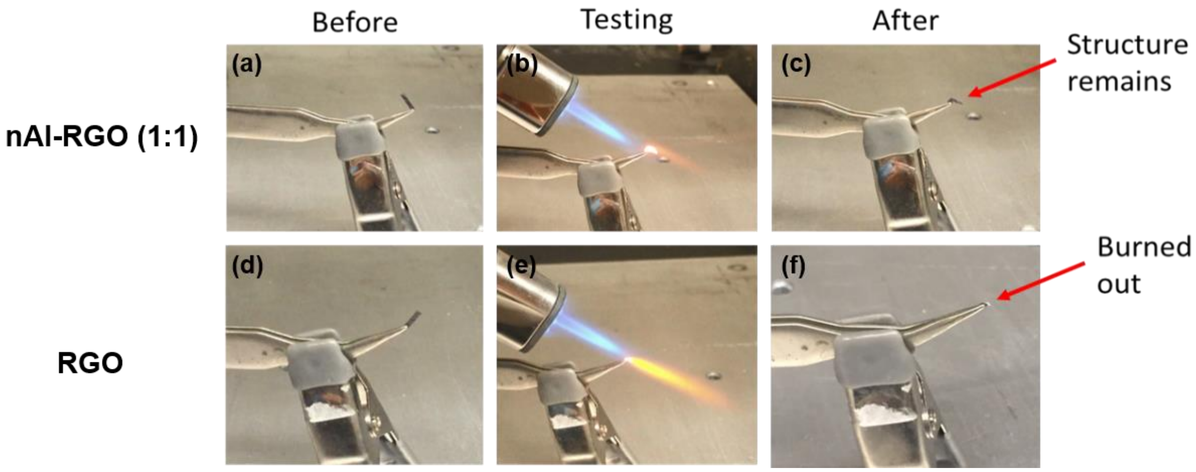

Supplementary Figure 14. A piece of nAl-RGO (1:1) and RGO film was heated with a butane torch.

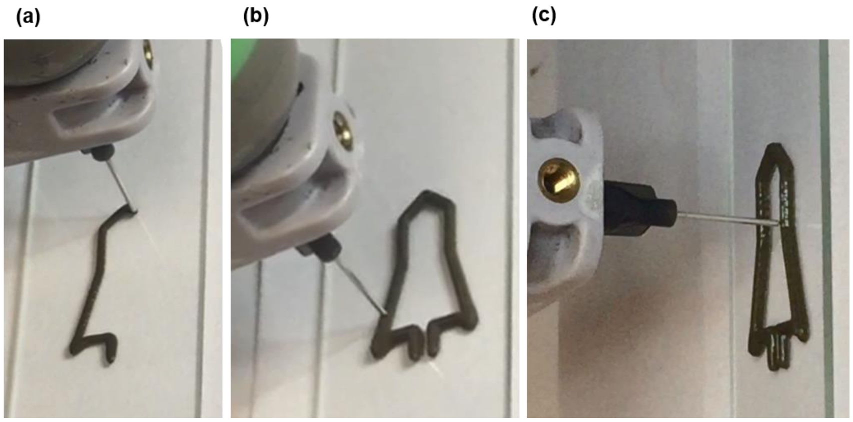

Supplementary Figure 15. 3D printing process of Al-GO pattern

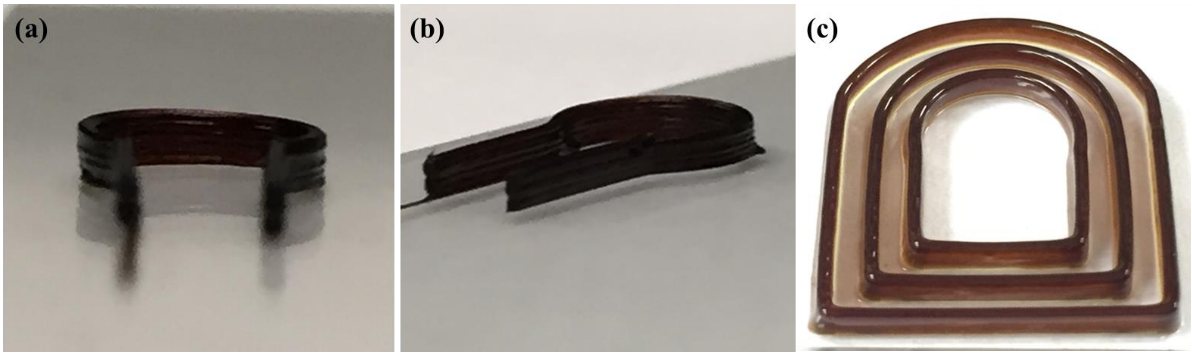

Supplementary Figure 16. Digital images showing various multilayered 3D printed GO based structure.

Supplementary Notes

Supplementary Note 1. Raman characterizations of nAl-RGO film.

During the high temperature treatment process, the intensity ratio of D peak to G peak (I_D/I_G) of nAl-RGO film always keeps a high value with applied current (Supplementary Fig. 4). The increasing current corresponding to the increasing temperature. Such a high value of I_D/I_G could be attributed to the high defects density in nAl-RGO film in the high temperature treatment process, which is strikingly different from the high temperature treated pure RGO film (Supplementary Fig. 5). The 2D peaks at 2680 cm^{-1} show up after high temperature treatment.

Supplementary Note 2. Morphology of nAl-RGO network with different process conditions.

The interior morphology of nAl-RGO is shown in Supplementary Fig. 6 after peeling off the surface, which exhibited uniform and high density Al nano particles distributed on graphene, indicating our Al nanoparticles are uniformly distributed all over the film. In addition, as shown in Supplementary Fig. 7, cross-sectional view of the nAl-RGO films presented a uniform Al nano particles distribution morphology on RGO layers, providing further evidence for our Al-RGO uniform nano composite.

In addition, our Al nanoparticles and RGO composite can be produced within 1 min to even ~ 10 ms. TEM images shown in Supplementary Fig. 8 is produced by increasing the current form 0 A to 1.05 A, immediately by pressing the button on Keithley 2400. Furthermore, we setup ultra-fast current pulse from 0 A to 1.05 A within 1 ms using Keithley 2400 program (Fig. 4a). The SEM images after heating the Al-RGO film (1050 µm long, 420 µm wide, and 4 µm thick) by 1 ms

current pluse are shown in Supplementary Fig. 9, which confirms that uniform and high density Al-RGO nanocomposite can be synthesized in an ultra-fast process.

In order to quantify the ultra-fast nature of the high temperature self-assembly of nano particles, it was imperative that the diagnostics should be able to resolve the sub millisecond heating time scales. In order to do so, the light emanating from the heated film was collected and sent into a 0.5 m spectroscope (Acton SP 500i), which had a 150 l/mm grating to disperse the light. The resulting spectrum was collected by a 32 channel PMT array interfaced with a high-speed data acquisition system (Vertilon IQSP 580). The data acquisition was done over the wavelength range of 500 – 800 nm (incorporating 24 channels of the PMT) and the system sensitivity was calibrated using a black body furnace in the range of 1200-1500 K. In this configuration (Supplementary Fig. 10), each channel collected light over a band of wavelength ~ 13 nm wide. The sample rate on the acquisition system was set at 10000 Hz, which produced a sample every 100 microseconds, sufficient to resolve the sub millisecond joule heating of the film. The spectrum was subsequently fit to the black body radiation, with temperature as a free parameter, using Planck's law to obtain the time resolved temperature profile.

Supplementary Note 3. Interaction energy between a foreign atom and a graphene with a slit defect

For a foreign atom located at (0, 0, d*)* near a graphene layer, its distance to a material point in graphene (*x, y*, 0) is given by $r = \sqrt{x^2 + y^2 + d^2}$.

The energy between the foreign atom and the material point in graphene due to vdW force is represented by a Lennard-Jones 6-12 potential,

$$V(r) = 4\epsilon\left(\frac{\sigma^{12}}{r^{12}} - \frac{\sigma^{6}}{r^{6}}\right) \quad (2)$$

where $\sqrt[6]{2}\sigma$ is the equilibrium distance between the foreign and carbon atoms and ϵ is the bond energy at the equilibrium distance.

Denote ρ as the carbon atom density per area of the graphene. The interaction energy between the foreign atom and an infinitesimal area of graphene $dxdy$ is then given by

$$\mathrm{dE}_{vdW} = \rho V(r) dxdy \tag{3}$$

Therefore, the interaction energy between the foreign atom located at (*x, 0, d*) and an infinitely large graphene with an infinitely long slit of width *w* along *y* direction can be computed by

$$\mathrm{E}_{vdW} = \int_{-x-w/2}^{\infty} \int_{-\infty}^{\infty} \rho V(r)\, dxdy + \int_{-x+w/2}^{\infty} \int_{-\infty}^{\infty} \rho V(r)\, dxdy. \tag{4}$$

The line plots in the right panel of Fig. 4a are obtained by evaluating Eq. 4 by numerical integration using the following parameters related to MD simulations: $\epsilon = \epsilon_{Al-C}$ = 0.0079 eV, $\sigma = \sigma_{Al-C}$ = 0.39495 nm, $d = \sigma$ = 0.39495 nm. For graphene, the atom density $\rho = \frac{4}{3\sqrt{3}l_0^2}$, where l_0 = 0.142 nm.

Supplementary Note 4. Carbothermic Reduction of Al_2O_3

At high temperatures, alumina can react with carbon through the overall reaction $2Al_2O_3 + 3C \rightarrow 4Al + 3CO_2$ to produced elemental Al in a process often referred to as carbothermic reduction. To illustrate the feasibility of this process under the conditions of our experiment, we used the equilibrium code CHEETAH 6.0 (using the JCZS product library) to calculate the most stable species at temperatures ranging from 1000 K to 3000 K. The reactants were set to Al_2O_3 and graphite (closest approximation to graphene available) in a 1:1 weight ratio and pressure was set at 0.001 atm as in the experiment. The resulting major species are plotted versus temperature in Supplementary Fig. 13. As can be seen, Al_2O_3 will start to decompose at 1700 K to produce Al vapor. This temperature is somewhat higher than what is produced in our system, however the

high surface area intrinsic to nanomaterials tends to cause processes to occur earlier. This also shows the production of aluminum carbide at some temperatures, however this is only to a small degree and was not found in any of the experimental XRD results. Instead, it seems that the produced Al vapor is trapped in the graphene matrix to produce the observed nanoparticles.

Supplementary Note 5. Flame Test of nAl-RGO film

To exhibit the safety of the produced composite, a butane torch was used in an attempt to burn the material. Supplementary Fig. 14 shows nAl-RGO (1:1) and RGO films heated for ~5 seconds responded similarly with the production of hot gases but exhibited no strongly violent. The RGO was brighter and was consumed in the process while the composite material remained somewhat intact. This result indicates that the RGO-nAl composite is at least as safe to handle as RGO, which is generally not considered hazardous.

Supplementary Note 6. 3D Printing of Al-RGO pattern

Supplementary Fig. 15 is a 3D printing process of the Al-GO with a rocket shaped pattern, conducted by a robotic 3D printer Fisnar F4200n. A high-concentrated Al-GO ink of 20 mg/ml in ethanol was prepared before printing for a stable and reliable flow via a 0.254 mm cylindrical nozzle. The printing process is at a horizontal speed of 50 mm/s with a pressure of 60 psi. After the Al-GO pattern is deposited onto a glass substrate, it was baked overnight at 60 °C followed by a thermal reduction at 500 °C in Ar/H_2 furnace for 1 hour. This printed Al-GO pattern can then be used to incorporate Al nano particles with rapid thermal annealing. Note that besides the above mentioned bake method to dry 3D printed pattern, freeze-dry was employed to demonstrate more complex 3D printed GO based structure (Supplementary Fig. 16).

Supplementary Methods

Preparation of Al-RGO film

a) Preparation of GO ink.

An improved Hummer's method was employed for the preparation of GO ink. The procedure flow is as follows. First, a suspended solution of a mixture of 1.5 g natural graphite flakes and 9 g of $KMnO_4$ in 200 mL acid of H_2SO_4/H_3PO_4 (volume ratio 9:1) was prepared. For better dispersion, the solution was heated to 50 ^oC while stirred continuously for 12 hours. After achieving a uniform composition, the solution was cooled down to room temperature before pouring onto 200 mL ice mixed with 3 mL H_2O_2. Subsequently, 100 mL 30% HCl solution with a DI water bath was used to wash away unwanted flakes. The resulting GO solution had a concentration of 2.5 mg mL^{-1} after diluting it in distilled water. To inspect the morphology of the obtained GO sheets, we dried several solution drops onto a silicon wafer. Supplementary Fig. 1 shows the optical microscopy images of the sheets. The resulting thickness is about 2 to 3 monolayers judging from the atomic force microscopy (AFM) images, as shown in Supplementary Fig. 1b.

b) Preparation of Al-GO solution

The Al powders ink was prepared by adding 30 mg of micro Al powders (99.5%, Sigma-Aldrich) into 12 mL ethanol, followed by 1 minute sonication. The concentration of the prepared Al powders solution is 2.5 mg/mL. The Al-GO solution was prepared by mixing the as prepared GO solution and Al powders solution with a weight of 1:1, followed by 1 minutes of shaking on a Tube Vertex Mixer and then 10 seconds of sonication. Thus, high quality Al-GO solution with

GO sheets and monodispersed Al powders can be obtained since the GO sheets serve as an excellent surfactant to disperse Al micro powders to form a uniform Al-GO suspension.

c) Preparation of Al-GO Film

Vacuum filtration: A freestanding Al-GO film (35 mm in diameter) was obtained by filtering 6 mL of the Al-GO solution through a 0.65 μm pore-sized membrane (Millipore, U.S.A). The film on membrane is dried in air. The Al-GO film can then be detached from the membrane naturally. The obtained Al-GO film with a thickness of around 4 μm was used for energetic application.

d) Reduction of Al-GO Film

Thermal pre-reduction of Al-GO film was carried out in a tube furnace under H_2/Ar (5%/95%) atmosphere, held at 300 ^oC for 10 min with a ramping rate of 5 ^oC/min.

e) Device geometry

A substrate for holding the free-standing Al-RGO film was prepared with glass slides, shown in Supplementary Fig. 2. Two cooper ribbons (2 mm wide, 20 mm long, 0.3 mm thick) were attached at each side of the glass mount, functioning as the connections to external circuit and heat sinks. Silver paste (SPI supplies) was applied to both ends of Al-RGO film as electrodes, forming an Ohmic contact to the later annealed film.

High temperature treatment

The high temperature annealing process was achieved by applying electrical current or voltage to the mounted Al-RGO film in a vacuum chamber. The system is shown in Supplementary Fig. 3, and the vacuum chamber is around 11 cm height and 17 cm in diameter. The glass mount is described in the above section of device geometry. To improve the accuracy of the spectrum

fitting with blackbody equation, the trench of the glass mount was painted black to avoid reflection.

Temperature measurement

Our spectral measurement system has been carefully calibrated by a NIST-traceable light source to ensure accuracy of the measurement. The effect of the glass window of the vacuum chamber has also been excluded. A multimode optical fiber with a diameter of 400 μm was aligned on top of the illuminating device and the sample irradiation intensity has been optimized before the spectrum was recorded.

The recorded emission spectrum of the Al-RGO device was then fitted to the Blackbody radiation equation to extract the *in situ* temperature of the film. The equation can be expressed as:

$$I_{\lambda}(\lambda,T)=\gamma\varepsilon_{gray}\frac{2hc^{2}}{\lambda^{5}\left[\exp\left(\frac{hc}{\lambda k_{B}T}\right)-1\right]} \quad (1)$$

where k_B is the Boltzmann constant, h the Planck constant, c the speed of light, and γ the scaling constant taking consideration of the unknown coupling factor between the Al-RGO sample illumination and the input of the optical fiber. ε_{gray} is a realistic range of emissivity. By a mathematical fitting of each curve from Fig. 2f to Eq. (1), the sample temperature can be extracted. Each fit curve (Figure 2f) is an average based on the four emissivity curves considered and the error bars denote the standard deviation.

Electrical measurement of Al-RGO film before and after high temperature treatment

The silver paste has provided for an Ohmic contact with low contact resistance. With a linear fit of IV curve extracted from the Keithley unit, the electrical conductivity of the Al-RGO film

before and after the high temperature treatment can be estimated (in Fig. 2d). The contact resistance is 2 Ω.

Self-assembly of Pd, Au, Si and Sn on RGO film

Pd-RGO film was made by mixing micro Pd powders (3 micron, 99.95%, from GoodFellow) and GO ink with a weight ratio of 1:3, followed by vacuum filtration and thermal reduction, which is similar to the above Al-RGO film preparation. The nPd-RGO, as shown in Fig. 3d, was self-assembled by heating the film at ~ 2000 K for 1 min.

Au-RGO film was made by mixing micro Au powders (10 micron, 99.9%, from Sigma-Aldrich) and GO ink with a weight ratio of 1:1, followed by vacuum filtration and thermal reduction, which is similar to the above Al-RGO film preparation. The nAu-RGO, as shown in Fig. 3e, was self-assembled by heating the film at ~ 1450 K for 1 min.

Si-RGO film was made by mixing nano Si powders (100 nanometer, 98%, from Sigma-Aldrich) and GO ink with a weight ratio of 1:1, followed by vacuum filtration and thermal reduction, which is similar to the above Al-RGO film preparation. The nSi-RGO, as shown in Fig. 3f, was self-assembled by heating the film at ~ 1550 K for 10 min.

Sn-RGO film was made by mixing micro Sn powders (10 micron, 99%, from Sigma-Aldrich) and GO ink with a weight ratio of 1:1, followed by vacuum filtration and thermal reduction, which is similar to the above Al-RGO film preparation. The nSn-RGO, as shown in Fig. 3g, was self-assembled by heating the film at ~ 1500 K for 40 min.
